# Testing the Functional and Phylogenetic Assembly of Plant Communities in Gobi Deserts of Northern Qinghai–Tibet Plateau

**DOI:** 10.3389/fpls.2022.952074

**Published:** 2022-07-18

**Authors:** Jianming Wang, Yin Wang, Mengjun Qu, Yiming Feng, Bo Wu, Qi Lu, Nianpeng He, Jingwen Li

**Affiliations:** ^1^School of Ecology Nature Conservation, Beijing Forestry University, Beijing, China; ^2^Key Laboratory of Ecosystem Network Observation and Modeling, Institute of Geographic Sciences and Natural Resources Research, Chinese Academy of Science, Beijing, China; ^3^Institute of Desertification Studies, Chinese Academy of Forestry, Beijing, China

**Keywords:** Gobi deserts, Qinghai–Tibet Plateau, assembly processes, functional traits, phylogeny

## Abstract

The mechanism governing plant community assembly across large-scale Gobi deserts remains unclear. Here, we inferred the roles of different assembly processes in structuring plant communities in the Gobi deserts of the Qinghai–Tibet Plateau by using a phylogenetic tree, and leaf and root traits. The functional and phylogenetic structures of 183 plant communities were assessed, and their distributions were linked with environmental gradients. Our results demonstrated that functional convergence was prevalent in most functional traits (75% of the traits) and accentuated when all traits were combined. The phylogenetic structure exhibited significant divergence. We observed the contrasting response of functional and phylogenetic assembly structures to environmental gradients. More importantly, we found that the shifts in the functional assembly along environmental gradients were trait-specific, with dominant roles of local factors, such as gravel coverage and soil attributes, in determining the distribution patterns of most traits. However, the distribution patterns of leaf P concentration (LPC), root N concentration (RNC), and root P concentration (RPC) were mainly driven by climatic factors. These results reveal that niche-based processes, such as abiotic filtering and weaker competitive exclusion, are the major drivers of species co-occurrence, which results in the widespread coexistence of phylogenetically distinct but functionally similar species within the Gobi plant community. Our findings could improve the understanding of plant community assembly processes and biodiversity maintenance in extremely harsh drylands.

## Introduction

Global changes are expected to substantially influence biodiversity and ecosystem functioning (Liu et al., 2018; Zellweger et al., 2019; Trisos et al., 2020). Hence, uncovering the fundamental mechanism underlying the plant assembly structure is crucial to understanding how plant communities respond to environmental changes (Enquist et al., 2015). Functional traits characterize the ecological strategies used by species to respond to environmental changes (DiAz and Cabido, 2001; Violle et al., 2007). Phylogenetic structure measures the evolutionary lineages among species within a community (Webb et al., 2002). Therefore, trait-based and phylogenetic analyses can help infer the assembly mechanisms governing plant communities (Cadotte et al., 2013; De Bello et al., 2017; Mugnai et al., 2022). However, the functional and phylogenetic structure exhibits distinct distribution patterns and is subjected to different controlling processes (Garcia-Giron et al., 2019; Wang et al., 2019). More importantly, the relationships among functional and phylogenetic diversity covary in different ways along environmental gradients (Bernard-Verdier et al., 2013; Purschke et al., 2013). Therefore, the approaches that combine trait-based and phylogenetic analyses have been employed by an increasing number of studies.

Plant assembly processes can be predominantly explained by niche and neutral theories (Chase and Myers, 2011; Hubbell, 2011). Niche theories focus on niche-based processes, such as abiotic filtering and biotic interactions (Swenson et al., 2012; Kraft et al., 2015). Abiotic filtering or weaker competitive exclusion can lead to functionally or phylogenetically convergent communities (Westoby and Wright, 2006; Mayfield and Levine, 2010; Backhaus et al., 2021), while biotic competition would result in the coexistence of phylogenetically or functionally dissimilar species (Macarthur and Levins, 1967; Cornwell and Ackerly, 2009; Bernard-Verdier et al., 2012). The neutral theory assumes that all individuals in plant communities are ecologically equivalent and that the plant community structure is determined by stochastic processes, such as birth and death of individuals, and extinction of species in a locality (Cadotte and Tucker, 2017; Perronne et al., 2017). Multiple assembly processes work together to govern plant communities, whereas their relative roles depend on spatial sales and habitat types (Lhotsky et al., 2016; Luo et al., 2019). For example, abiotic filtering would be more important in more stressful environment, resulting plant functional convergence (De Bello et al., 2009; Kraft and Ackerly, 2010). By contrast, biotic interaction could dominate plant communities in a stable environment, and plant communities would show functional divergence (Mayfield and Levine, 2010).

More importantly, variations in environmental factors can alter the balance between different assembly processes (Lhotsky et al., 2016; Ding et al., 2019; Wang et al., 2021a). Depending on ecosystem types and inquiry scales, the plant community assembly is influenced by various environmental factors such as elevation, temperature, and soil attributes (Bernard-Verdier et al., 2012; Luo et al., 2019; Wang et al., 2021a). Therefore, exploring the influence of environmental factors on plant phylogenetic and functional trait distribution may provide new insights for predicting how plant community assembly responds to future climate changes (Enquist et al., 2015; De Pauw et al., 2021). However, how multiple environmental factors jointly drive the variation in the assembly processes of plant communities across large-scale Gobi deserts remains unclear.

The Qinghai–Tibet Plateau (TP), referred to as “the world's roof”, is mainly characterized by intense solar radiation, heavy water deficit, and nutrient limitation. These environmental regimes may lead to the unique ecological strategy and coexistence mechanism of plant species in the Gobi deserts of TP (Guo et al., 2011). Recently, the rate of climate warming on the TP has been more than twice the global average (Ma et al., 2017; Yao, 2019). Therefore, testing the relative roles of the different processes in shaping plant functional and phylogenetic structure in the Gobi deserts of TP may provide new insights into the mechanisms underlying the generation and maintenance of plant diversity under global change. Indeed, the plant community assembly and its determinants have been well examined in steppe and meadow ecosystems of TP (Wang et al., 2021a,b). However, few studies to date have focused on elucidating the assembly processes that determine plant functional and phylogenetic structures across the Gobi deserts of TP.

This study mainly aimed to (1) compare the variation in plant functional and phylogenetic distribution along environmental gradients and (2) whether and how environmental factors drive the variation in plant assembly processes. We selected 183 plant communities from the typical Gobi deserts of TP and assessed functional traits and phylogenetic trees. We examined the following hypotheses: (1) the plant functional and phylogenetic structure was significantly convergent in harsh Gobi deserts and (2) multiple environmental factors jointly drive the plant assembly processes, whereas water availability plays a more important role.

## Methods

### Study Sites

**Figure 1:**
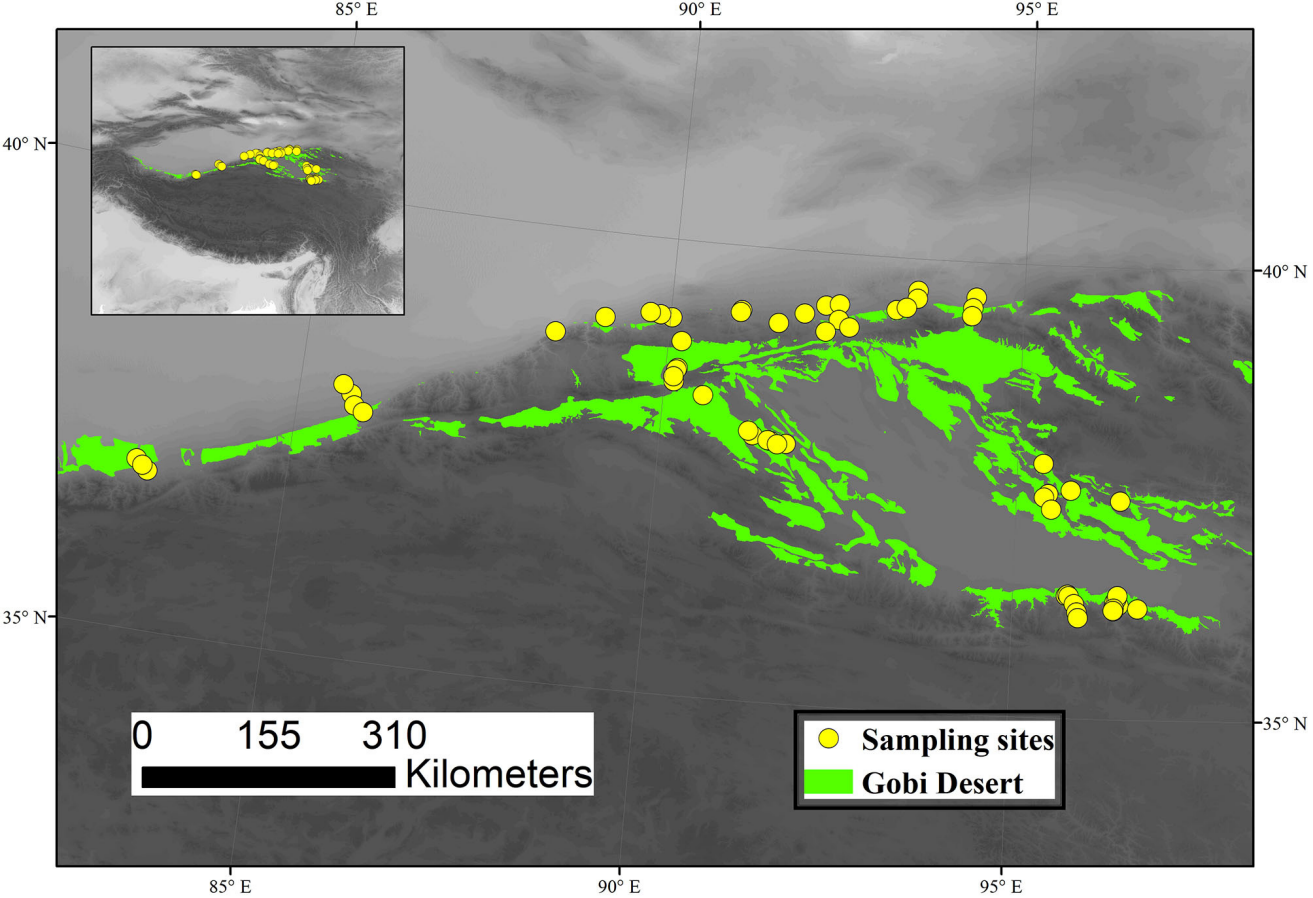
Distribution of sampling sites in Gobi deserts of northern Qinghai–Tibet Plateau.

Gobi deserts are the main ecosystem types in the northern Qinghai–Tibet Plateau, which has a total area of ~84, 928 km^2^. The climate of the study area is typical plateau temperate, changing from extremely arid to arid, with strong spatial variability in precipitation and temperature. The vegetation types are mainly dominated by shrubby desert (SHD), dwarf semi-arboreous desert (DSAD), semi-shrubby and dwarf semi-shrubby desert (SHDSD), and succulent holophytic dwarf semi-shrubby desert (SHSHD). We selected 61 sites from the typical Gobi deserts region in northern Qinghai–Tibet Plateau during the peak of the growing season (July-August) of 2015, which covered major climatic zones and vegetation types (Figure 1). Specifically, 4 sites of DASD, 23 of SHD, 31 of SHDSD, and 3 of SHSHD were selected in this study. DASD was mainly dominated by *Haloxylon ammodendron*; SHD was mainly dominated by *Ephedra przewalskii, Sarcozygium xanthoxylon, Calligonum mongolicum, Tamarix ramosissima* and *Nitraria tangutorum*; SHDSD was mainly dominated by *Reaumuria songarica, Sympegma regelii, Ceratoides latens* and *Reaumuria kaschgarica*.

### Field Survey and Sampling

At each site, three 10 m × 10 m plots were randomly established along a 1-km transect under representative landscape and dominant vegetation, and geographic coordinates and elevation of each plot were recorded with GPS. All plant species compositions and abundance were identified and recorded at the plot level. Gravel coverage (Gravel) by visually estimating the percent of a plot occupied by the vertical projection of all gravel onto the ground. At each plot, 15 soil cores (0–10 cm) were randomly collected and subsequently mixed into a composite sample.

Approximately 30 mature but non-senescent leaves with little damage were collected from different locations at each site to determine leaf traits. Specific leaf area (SLA, cm^2^/g) was measured by assessing the ratio of the leaf area (LA) to its oven-dried mass. For fine-root (diameter ≤ 2 mm) sampling was conducted following the protocol described by Guo et al. (2011). Specifically, for woody species, we first removed the surface soil near the plant basal stem and then traced the intact root system to the lateral root clusters. For herbaceous species, root samples were obtained by separating root systems from whole plants. Given that desert plants usually have limited fine root amounts, root samples were collected from at least 20 individuals. The ratio of root length (RL) to dry mass was used to calculate specific root length (SRL, m/g). The leaf N concentration (LNC, mg/g) and root N concentration (RNC, mg/g) was determined by dry combustion using an elemental analyzer (FLASH2000 CHNS/O, Thermo, American). Leaf P concentration (LPC, mg/g) and root P concentration (RPC, mg/g) were measured using colorimetry after digestion with H_2_O_2_-H_2_SO_4_.

Finally, eight key leaf and root traits: SLA, LA, LNC, LPC, SRL, RL, RNC, and RPC were used in this study. LA represents determines the size of the photosynthetic surface, while SLA can reflect carbon assimilation and growth rate of plants (Cornelissen et al., 2003). LNC can reflect the photosynthetic rate, plant growth, and survival, while LPC represents the nutritional quality and plant growth (Wright et al., 2004; Pérez-Harguindeguy et al., 2013). RL can reflect the difficulty in extracting fine roots from the soil, and the enormous length these roots can attain, while SRL represents the nutrient and water absorption, root lifespan, and relative growth rate (Pérez-Harguindeguy et al., 2013). RNC and RPC can reflect root nutrient uptake efficiency (Freschet et al., 2017). Therefore, we selected these eight traits to infer functional assembly.

### Environmental Variables

Soil total nitrogen content (TSN) and organic carbon content (TOC) was measured using the Kjeldahl procedure and K2Cr2O7 oxidation method, respectively. Soil moisture (SM) was measured gravimetrically, and soil pH was determined by 1:2.5 (v/v) soil water aqueous extract. For climatic factors, we obtained the data of precipitation seasonality (PS), mean annual precipitation (MAP), temperature seasonality (TS), and mean annual temperature (MAT) from the Worldclim global climate database (https://www.worldclim.org/data/index.html). Hence, nine environmental variables (local factors: Gravel, TSN, TOC, SM, pH; Climatic factors: MAP, PS, MAT, TS) were used in this study.

### Phylogenetic Tree Reconstruction

All species names were standardized following The Plant List within “plantlist” package. We identified a total of 44 species that could be classified into 15 families and 35 genera (Supplementary Table S1). After that, the species names were linked with those in megaphylogeny using phylo.maker function within V.PhyloMaker package, and scenario 3 approach to add species to the phylogeny (Jin and Qian, 2019). Scenario 3 adds missing taxa (e.g., genus or species) to the phylogeny within the taxa with known branch lengths (Jin and Qian, 2019). Finally, a phylogenetic tree of all 44 species was reconstructed under scenario 3 (Supplementary Figure S1), which is similar to the approach implemented in Phylomatic and BLADJ.

### Phylogenetic Signal of Traits

Phylogenetic signals of each functional traits were quantified using Blomberg's *K* to infer the relationship between functional and phylogenetic structure (Blomberg et al., 2003). Significant phylogenetic signals indicate the closely related species have similar functional traits, and functional and phylogenetic structures showed similar patterns. The significances of Blomberg's *K* values were assessed by comparing to null distributions by shuffling species labels at the tip of the phylogeny 999 permutations. Additionally, all six functional traits were log-transformed before analysis (Luo et al., 2019).

### Phylogenetic and Functional Structure

Mean pairwise distances (MPD) of functional trait of all species within the community was used to determine the functional structure, and MPD was assessed for each of the six individual functional traits as well as for six of the traits combined. MPD is the mean functional and phylogenetic distance among all pairs of species within a community, which is widely used to infer community assembly structure in previous studies (Luo et al., 2020; Wang et al., 2021b). Meanwhile, the phylogenetic structure was determined as the MPD of species' phylogenetic relatedness. Both functional and phylogenetic MPD were calculated using the Picante package in R. Specifically, principal components analysis (PCA) was conducted with the vegan package to reduce functional trait data redundancy. Trait PCA axes were used to calculate functional structure. The standardized effect size (SES) of functional and phylogenetic MPD was calculated using the null model approach to infer the functional and phylogenetic assembly mechanisms. A total of 999 null communities were generated by randomly shuffling the species names at the tips of the functional and phylogenetic trees. SES of MPD (SES.MPD) was assessed using the following formula:


$$\begin{array}{l} SES\;\cdot \;MPD\;=\;\frac{MPD_{obs}\;-\;mean\left(MPD_{null}\right)}{sd\left(MPD_{null}\right)} \end{array}$$

where MPD_*obs*_ indicates the observed MPD values, mean (MPD_*null*_), and sd (MPD_*null*_) indicates the mean and standard deviation value of 999 null communities, respectively. Negative SES.MPD values indicate the phylogenetic and functional convergence, whereas positive SES.MPD values indicate the phylogenetic and functional divergence. Given the non-normality of data, the significant deviations of functional and phylogenetic MPD from null expectations (SES = 0) were tested using the Wilcoxon test. SES was significantly different from 0 mean that niche-based processes dominated the plant communities.

### Statistical Analysis

All data of functional and phylogenetic structure and environmental variables were standardized (average = 0 and SD = 1). The major drivers of the phylogenetic and functional structure were determined by stepwise multiple regressions (SMR). The relationship between phylogenetic/functional structure and the individual environmental variable was examined by linear regression. All variables were subjected to forward-selection until *p* < 0.05 for all explanatory variables. To avoid the strong collinearity among variables, we removed the variables following the criterion of variance inflation factor greater than 3. Finally, hierarchical partitioning was applied to explore the independent effect of each variable on community assembly, using hier.part package (Walsh et al., 2003). All analyses were carried out in R 3.6.3 (R Development Core Team, 2020).

## Results

### Phylogenetic Signals of Functional Traits

Among eight functional traits, only LA and RL showed a statistically significant phylogenetic signal (Table 1), whereas the six other functional traits did not. Meanwhile, Blomberg's *K* of LA and RL were obviously <1, indicating the weaker phylogenetic signal than expected by Brownian motion model for trait evolution. These results may indicate that evolutionary history or phylogenetic relationships had weak influence on functional traits.

**Table 1:** Phylogenetic signal of plant functional traits.

**Functional trait**	**Blomberg's *K***	***P***
Leaf nitrogen concentration (LNC)	0.10	0.28
Leaf phosphorus concentration (LPC)	0.10	0.31
Specific leaf area (SLA)	0.08	0.55
Leaf area (LA)	**0.43**	**0.03**
Root nitrogen concentration (RNC)	0.17	0.11
Root phosphorus concentration (RPC)	0.07	0.68
Specific root length (SRL)	0.19	0.08
Root length (RL)	**0.41**	**0.02**

*Values in bold highlight the significant signal*.

### Functional and Phylogenetic Assembly Structure

**Figure 2:**
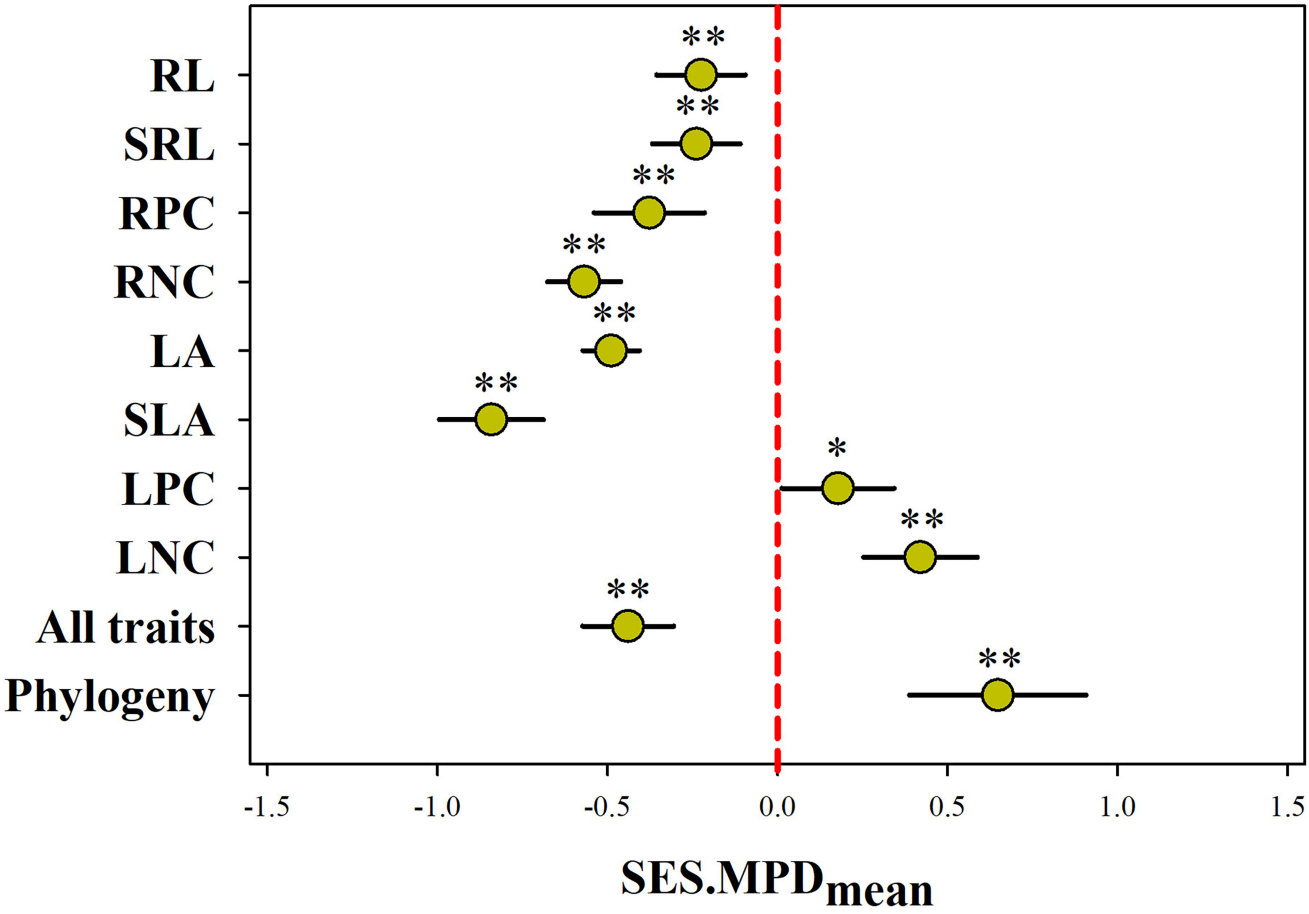
Plant phylogenetic and functional structure assessed using the mean (mean ± 95% confidence) of standardized effect size of mean pairwise distances (SES.MPD) across 183 communities. A negative value indicates phylogenetic and functional convergence, while positive value indicates phylogenetic and functional divergence. SLA, specific leaf area; LA, leaf area; LNC, leaf N concentration; LPC, leaf P concentration; SRL, specific root length; RL, root length; RNC, root N concentration; RPC, root P concentration; all traits, all traits combined; **, *p* < 0.01; *, *p* < 0.05.

Null model and Wilcoxon test analysis together showed the average SES.MPD for most functional trait was significantly less than expected by chance across all communities, except for LNC and LPC (*p* < 0.001; Figure 2). In contrast, the average SES.MPD for LNC and LPC was higher than expected by chance across all communities (*p* < 0.05). Meanwhile, the average SES.MPD for all of the traits combined was also significantly less than expected by chance across all communities (*p* < 0.001). However, we found the average phylogenetic SES.MPD was significantly larger than the zero value (*p* < 0.01).

### Variation in Functional and Phylogenetic Assembly Along Environmental Gradients

**Figure 3:**
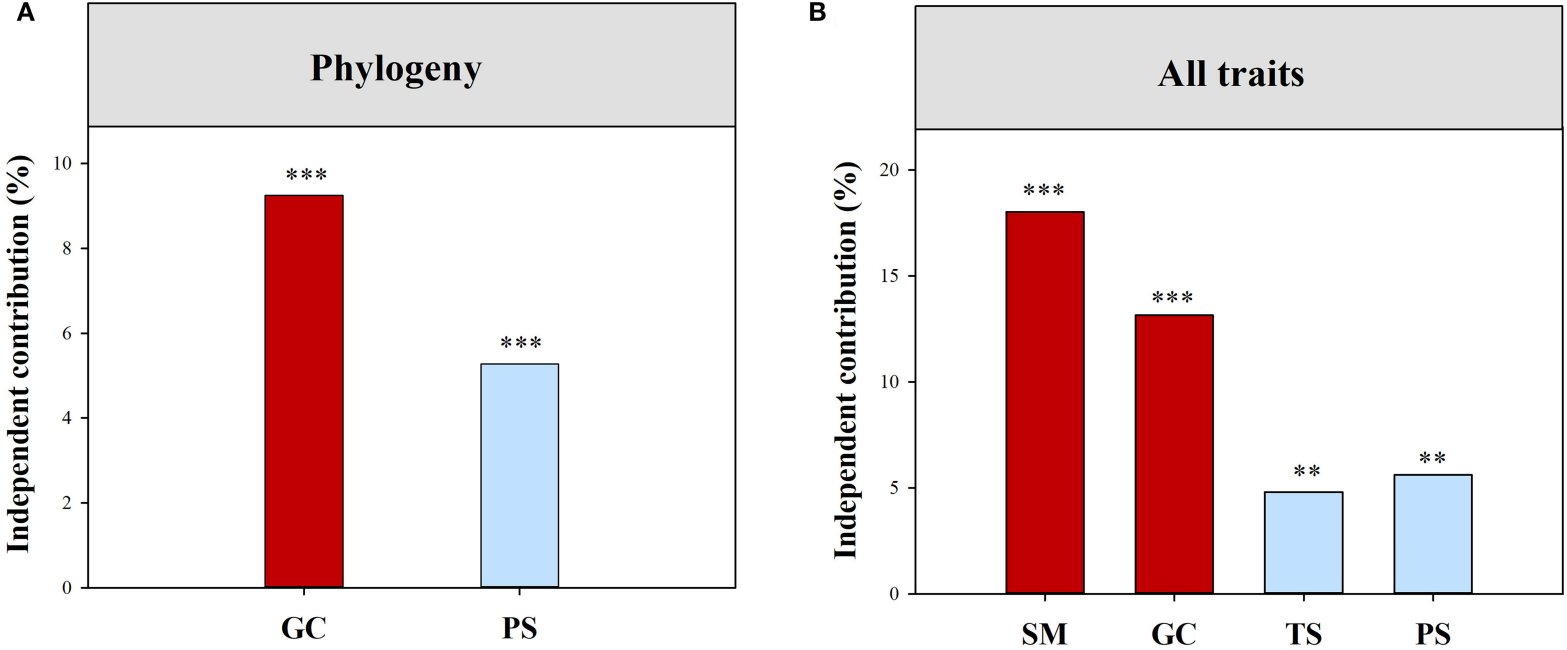
Independent influence of major variables on the variations in phylogenetic **(A)** and functional **(B)** structure. All traits, all traits combined; SM, soil moisture; PS, precipitation seasonality, PS; TS, temperature seasonality; GC, gravel coverage. ***, *p* < 0.001; **, *p* < 0.01.

**Figure 4:**
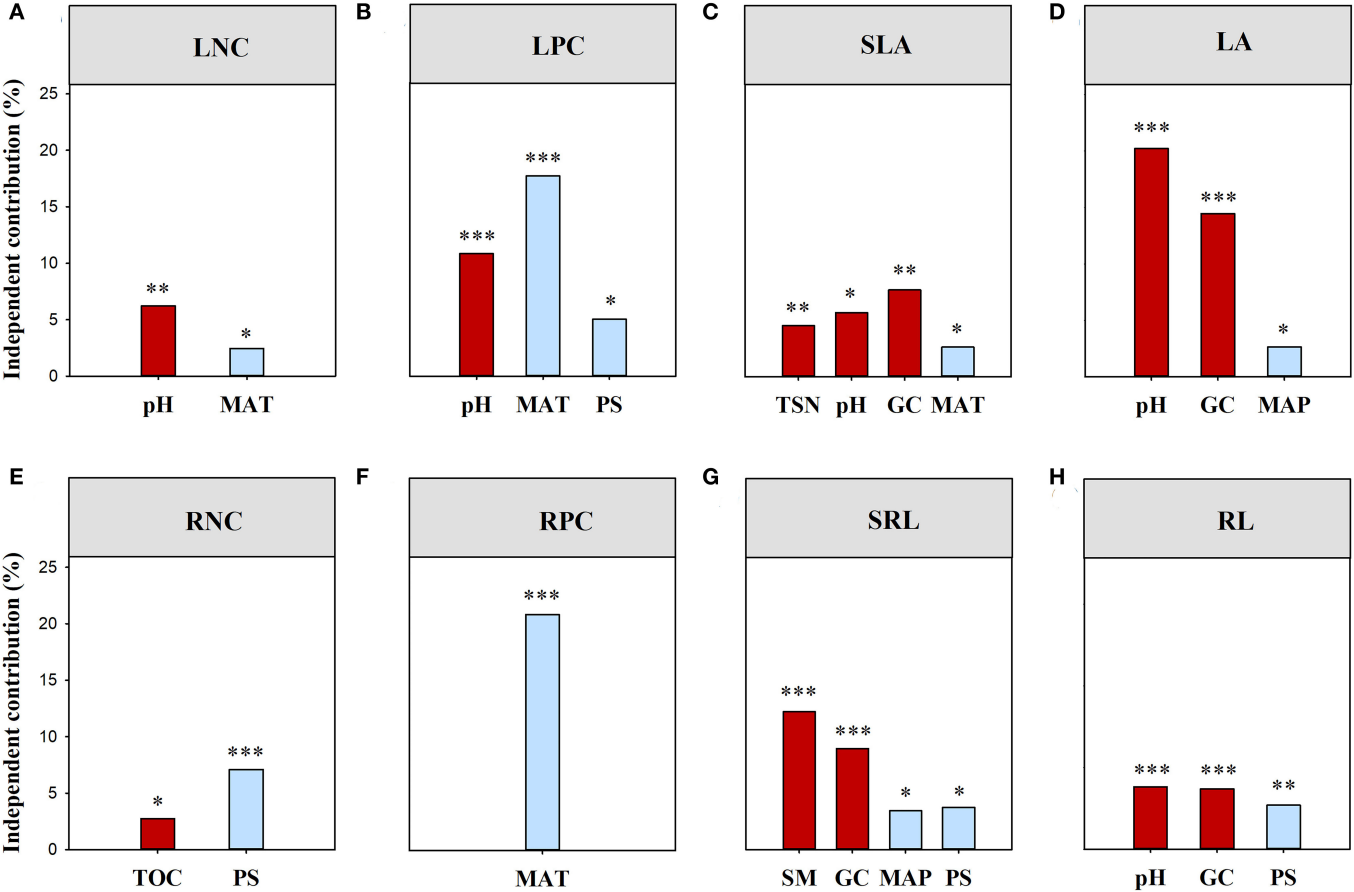
Independent influence of major variables on the variations in the standard effect size of MPD for the individual trait. MPD, mean pairwise distances. TSN, soil total nitrogen content; TOC, soil organic carbon content; SM, soil moisture; PS, precipitation seasonality; MAP, mean annual precipitation; TS, temperature seasonality; MAT, mean annual temperature; All traits, all traits combined; LNC, leaf nitrogen concentration; LPC, leaf phosphorus concentration; SLA, specific leaf area; LA, leaf area; RNC, root nitrogen concentration; RPC, root phosphorus concentration; SRL, specific root length; RL, root length. ***, *p* < 0.001; **, *p* < 0.01; *, *p* < 0.05.

Climatic and local habitat factors together explained 14.04% and 40.26% of the total variations in functional (all of the traits combined) and phylogenetic SES.MPD, respectively (Supplementary Table S2). The variation in functional (all of the traits combined) SES.MPD was mainly explained by SM, followed by Gravel, TS, and PS (Figure 3). However, Gravel and PS together drove the variation in phylogenetic SES.MPD (Figure 3). Moreover, climatic and local habitat factors together explained 7.67%-37.84% of the total variation in SES.MPD for eight functional traits (Supplementary Table S2; Figures 4A–H). The variations in the SES.MPD for LPC, RNC, and RPC was more influenced by climatic factors than local habitat factors, while those of SLA, LA, SRL and RL were more strongly related to local habitat factors (Figures 4A–H).

**Figure 5:**
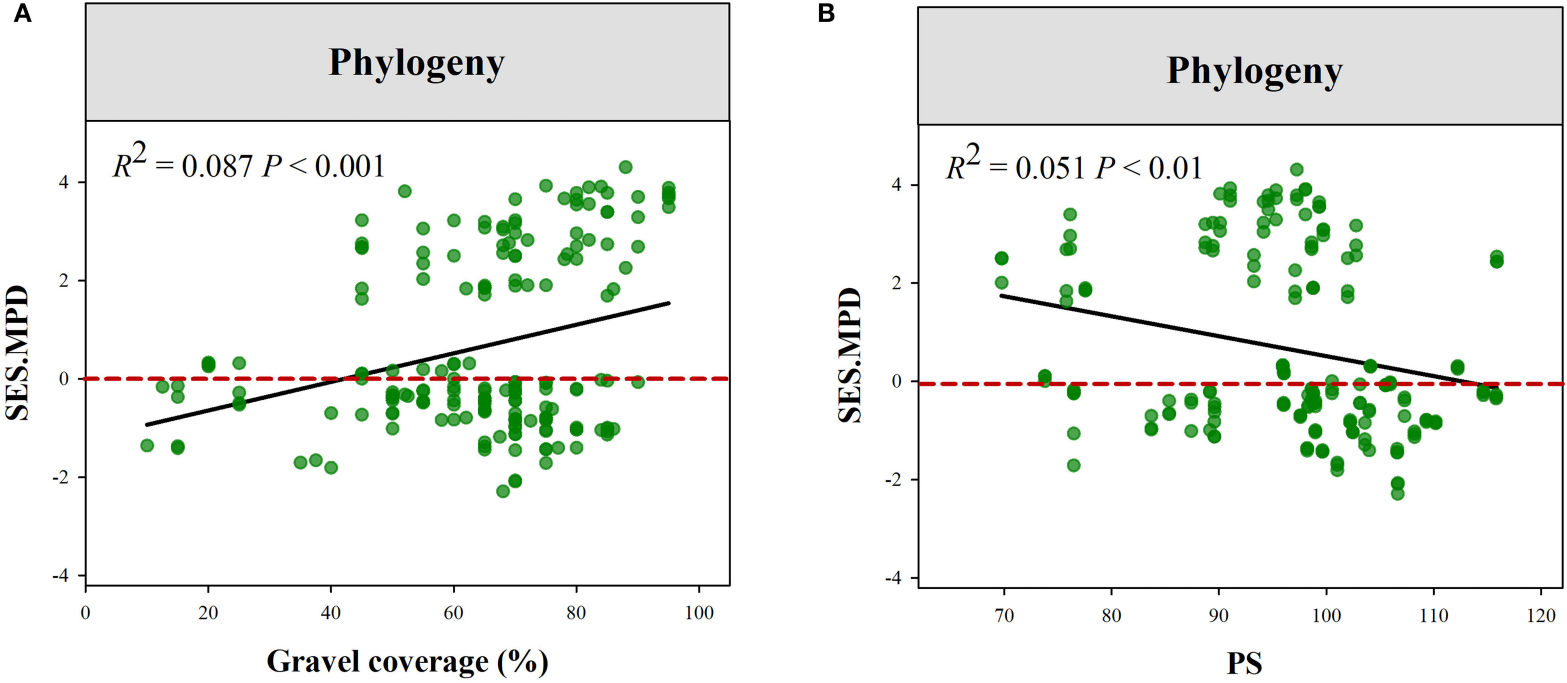
Variations in the standard effect size of phylogenetic MPD along the gradients of Gravel **(A)** and PS **(B)**. PS, precipitation seasonality; GC, gravel coverage.

**Figure 6:**
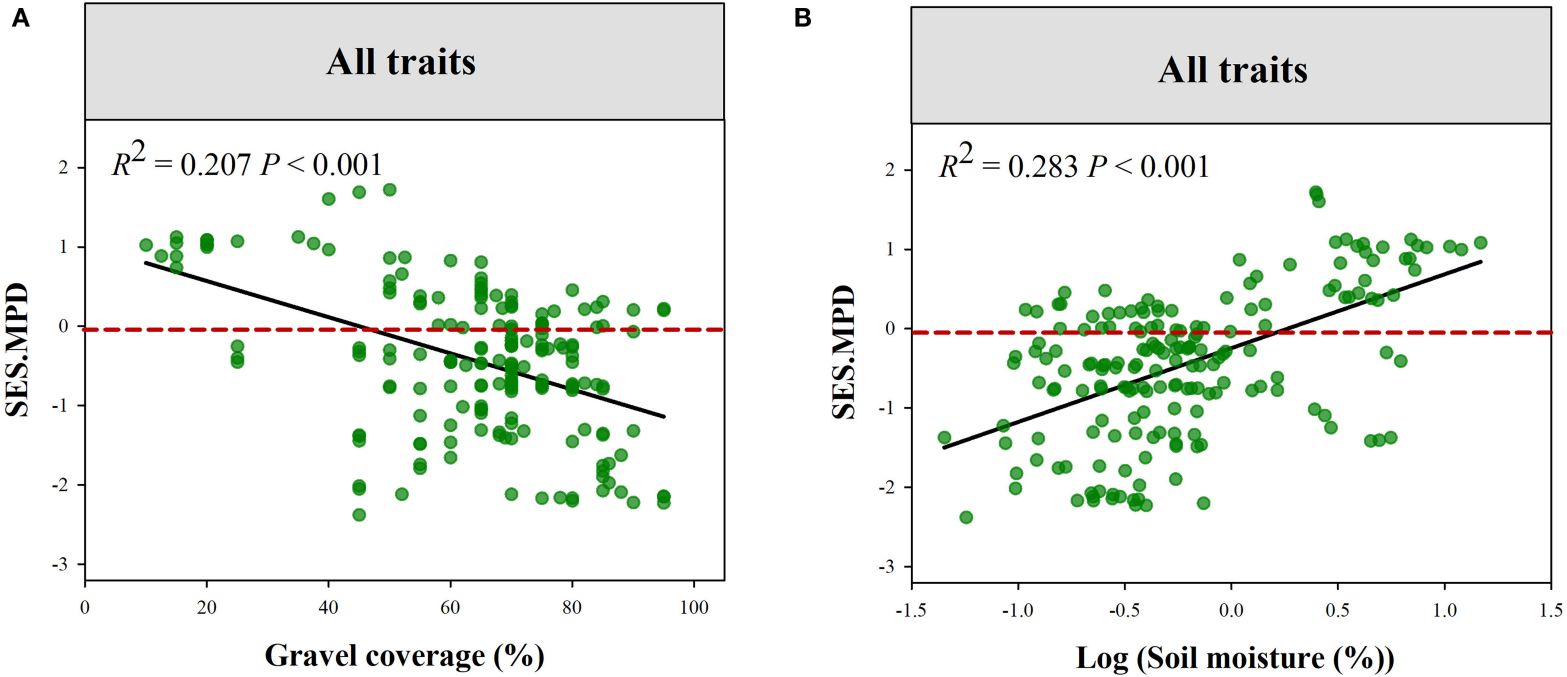
Variations in the standard effect size of functional MPD (all traits combined) along the gradients of Gravel **(A)** and SM **(B)**. GC, gravel coverage; SM, soil moisture.

The plant phylogenetic structure was more divergent in high Gravel or low PS gradient (Figure 5). Plant functional structure (all of the traits combined) was more convergent at low SM and PS or high Gravel and TS gradient (Figure 6, Supplementary Figure S2). Moreover, we observed that eight functional traits respond to climatic and local factors differently (Supplementary Table S2, Supplementary Figure S3–S10). For instance, the community structures of LNC and LPC were more convergent at high pH gradient, while those of SLA, LA, and RL were more convergent at low pH gradient. Additionally, the community structures of LNC and LPC were more convergent at high MAT gradient, while those of SLA and RPC were more convergent at low MAT gradient.

## Discussion

### Difference Between Plant Functional and Phylogenetic Distribution Patterns

Phylogenetic information can help elucidate the evolutionary relationships among co-existing species (Webb et al., 2002). Phylogeny could reflect important unmeasured traits or complex ecological strategies not easily captured by functional traits, such as plant–*Mycorrhiza* and plant–pathogen interactions (Gilbert and Webb, 2007; Cadotte et al., 2013; Montesinos-Navarro et al., 2015). Therefore, exploring the relationship between phylogenetic conservatism and trait evolution contributes to better inferring community assembly mechanisms (Purschke et al., 2013; Mugnai et al., 2022). When plant functional traits exhibited significant phylogenetic signals, plant functional and phylogenetic diversity generally should show similar patterns (De Bello et al., 2017). However, the relationships between functional and phylogenetic distribution patterns vary across spatial scales and habitat types (Purschke et al., 2013; Wang et al., 2019). Similarly, our study observed that eight functional traits exhibited weak phylogenetic signals, indicating that Gobi plants have weak phylogenetic conservatism result of the influence of local adaptation to the Gobi harsh environment (Shigyo et al., 2017). The *K* values of functional traits close to zero may reflect the adaptive convergence processes of Gobi plants driven by similar selection, especially for drought tolerance, which may be better captured by an Ornstein–Uhlenbeck (OU) process of trait evolution (Butler and King, 2004). Therefore, our findings imply that distant relatedness could be less functionally different because plants are restrained by desert environmental stress toward an optimal trait value (Butler and King, 2004), which in turn could possibly result in strong convergence among Gobi plant species. Hence, plant communities exhibited functional trait convergence (SES.MPD of all of the traits combined <0) and phylogenetic divergence (phylogenetic SES.PMD >0). In harsh Gobi deserts, convergence in functional traits, such as SRL and SLA, may also decrease the potential advantages in competitive ability (Luo et al., 2016), thereby promoting species coexistence. Together, we highlight that phylogenetic diversity cannot be simply used as the proxy for functional diversity; therefore, combining functional and phylogenetic approaches is necessary to accurately determine the Gobi plant community assembly.

### Niche-Based Processes Drive Plant Functional and Phylogenetic Assembly

The balance between different assembly processes determines the relative strength of convergence and divergence for trait and phylogeny. The dominance of neutral processes will induce a random community structure (Kraft and Ackerly, 2010). Stress-dominance hypothesis believes that abiotic filtering would dominate community assembly and leads to functional phylogenetic convergence in a more stressful habitat, while biotic interaction, such as competition exclusion, plays a major role and resulting in divergence in a more favorable environment (Schöb et al., 2013; Coyle et al., 2014). In this study, we observed that most traits, as well as all the traits combined exhibited functional convergence, consistent with the findings of Chinese grasslands (Wang et al., 2021a). This may suggest that abiotic filtering drives the Gobi plant community's functional structure. The environmental filtering hypothesis posits that abiotic filtering chooses species with similar trait values within communities (Kraft et al., 2015; Šímová et al., 2015). Abiotic filtering would be stronger in harsher habitats, thereby further reducing functional trait diversity (De Bello et al., 2009; Kraft and Ackerly, 2010). A harsh and homogeneous Gobi-desert environment would filter species toward the optimal trait value, allowing them to cope with the strong abiotic stress and resulting in functional convergence (Keddy, 1992; Grime, 2006). However, the weaker competitor hypothesis also believes that relatively weaker competitive species would be excluded by relatively higher competitive species and can result in trait convergence (Mayfield and Levine, 2010). Additionally, our results partly support the importance of competitive exclusion, that is, LNC and LPC showed functional divergence, which is similar to the findings on desert steppe (Wang et al., 2021b). Overall, we highlight that multiple niche-based processes, such as abiotic filtering and weaker competitive exclusion, determine plant functional assembly.

Our results reveal that phylogenetic structure displayed significant divergence, which further supports the dominance of niche-based processes in Gobi deserts. We identified a total of 44 species of species that could be classified into 15 families and 35 genera (Supplementary Table S1). Hence, plant species in the study area have relatively more distant relatedness. For example, as the Gymnosperm plants, *Ephedra przewalskii* is present in more than 60 plant communities and has great distant relatedness with Angiosperm species. However, *E. przewalskii* shares similar functional traits with dominant angiosperm species, such as *Calligonum mongolicum, Nitraria sphaerocarpa, Gymnocarpos przewalskii*, and *Reaumuria kaschgarica*. Given the trait convergent evolution and distant relatedness among species, abiotic filtering and weaker competitive exclusion would select species with similar traits but distant relatedness into the community, leading to trait convergence but phylogenetic divergence among co-occurring species within the Gobi community. Together, this finding revealed that niche-based processes, such as abiotic filtering and weaker competitive exclusion, can result in the discrepant patterns of functional and phylogenetic distribution in Gobi deserts.

### Climatic and Local Habitat Factors Jointly Mediate the Relative Strength of Functional and Phylogenetic Convergence and Divergence

The relative strength of functional/phylogenetic convergence and divergence vary along environment gradients (Purschke et al., 2013; Luo et al., 2019). However, plant functional and phylogenetic distributions were driven by a wide range of environmental factor (López-Angulo et al., 2018; Backhaus et al., 2021; Catano et al., 2021). For example, trait convergence was mainly driven by temperature on the Tibet Plateau, whereas water availability governs trait convergence on Mongolian Plateau (Wang et al., 2021a). This study found that the distribution patterns of phylogeny and most traits were mainly influenced by local habitat factors, such as gravel coverage and soil condition, rather than climate. Similarly, a study on dryland reported that soil and topographic factors exhibited a more important influence on functional and phylogenetic diversity (Wang et al., 2019). Water availability plays a fundamental role in shaping ecosystem function and biodiversity (Fernandez-Going et al., 2013; Wang et al., 2014). However, the amount of water available to plants was not simply dependent on climatic factors (Zhang et al., 2017). Soil attributes are key determinants of the growth and distribution of plant species in drylands (Maestre et al., 2003). In Gobi deserts, gravel coverage could mediate the water availability for plant uses by affecting water infiltration and evapotranspiration (Unger, 1971). For example, we observed a negative correlation between gravel coverage and soil moisture (Supplementary Figure S11). Additionally, local discrete vegetation patches could alter the spatial heterogeneity of water and nutrients supply by generating “fertile islands” (Okin et al., 2004; De Graaff et al., 2014). Therefore, local factors can largely determine the functional and phylogenetic assembly in Gobi deserts. Whether the interactions between climatic and local factors can drive functional and phylogenetic distribution should be determined because this might lead to difficulty in quantifying their individual effects precisely (López-Angulo et al., 2018). Partly supporting the finding on United States drylands (Butterfield and Munson, 2016), we also found that climatic factors, such as mean annual temperature, were the best predictor of distribution of LPC and RPC. Meanwhile, we found that the distribution patterns of eight traits were influenced by different local habitat factors. These findings support the viewpoint that response of plant functional assembly structures to environmental gradients were trait-specific (Le Bagousse-Pinguet et al., 2017).

A previous study reported that plant functional and phylogenetic convergence showed a trend decreased along the sand desertification gradient (Wang et al., 2021b). The phylogenetic and functional structure of tree communities also showed similar shifts along a climatic gradient in Yulong Mountain (Luo et al., 2019). However, the shift in phylogenetic and functional structure along environmental gradients is not always accordant (Purschke et al., 2013; Cadotte et al., 2019). Our study demonstrated the contrasting shift in phylogenetic and functional structure along environmental gradients. For example, phylogenetic structure tended to be convergent in low gravel coverage habitat and divergent in higher gravel coverage habitat. However, functional structure exhibited an opposite change trend along gravel coverage gradients. Additionally, we also found that the functional convergence tends to decrease along soil moisture. In the study region, *E. przewalskii* belonging to Gymnosperm plants mainly occurred in more stressful environments, such as higher gravel coverage and lower soil moisture. Relatively lower environmental stress in low gravel coverage habitat leads to more functional distinct species, such as shrub and herbaceous species, that can co-occur, leading to functional divergence. However, stronger environmental stress would reduce the tolerable range for species growth and filter stress-avoidant species in high gravel coverage habitats (De Bello et al., 2009; Kraft and Ackerly, 2010). As a result, plant communities in these habitats were dominated by few functional similarly and stress-tolerant species. Hence, plant communities showed functional convergence but phylogenetic divergence in these stressful habitats. This finding suggests that particular species pool and trait convergent evolution lead to different responses of functional and phylogenetic structures to environmental stress.

## Conclusion

This study elucidates the assembly mechanism of plant community in large-scale Gobi deserts of Qinghai–Tibet Plateau by using eight functional traits and a phylogenetic tree. Our results demonstrated that niche-based processes, such as abiotic filtering and weaker competitive exclusion, are the major driver of species co-occurrences, which lead to the widespread coexistence of phylogenetically distinct but functionally similar species. More importantly, the shifts in the functional assembly along environmental gradients were trait-specific, with dominant roles of local habitat factors, such as gravel coverage and soil attributes, in determining distribution patterns of LNC, SLA, LA, SRL, and RL. The distribution patterns of LPC, RNC, and RPC were mainly driven by climatic factors. We also observed contrasting responses of functional and phylogenetic assembly to environmental gradients. Along the environmental stress gradient, functional convergence tended to increase, whereas phylogenetic divergence tended to increase. Our findings could reinforce the understanding of the generation and maintenance of plant biodiversity in extremely harsh drylands.

## Data Availability Statement

The original contributions presented in the study are included in the article/Supplementary Material, further inquiries can be directed to the corresponding author/s.

## Author Contributions

JW and JL conceived the project idea and led the data collection. JW, YW, BW, and JL participated in the transect investigation. JW and MQ implemented the analyses and writing. YF and QL helped with the data analysis. NH and JW revised the article. All authors discussed the results and contributed significantly to the final manuscript.

## Funding

This work was supported by the National Natural Science Foundation of China (32001186 and 31971538).

## Conflict of Interest

The authors declare that the research was conducted in the absence of any commercial or financial relationships that could be construed as a potential conflict of interest. The handling editor RZ declared a shared parent affiliation with the authors YF, BW, and QL at the time of review.

## Publisher's Note

All claims expressed in this article are solely those of the authors and do not necessarily represent those of their affiliated organizations, or those of the publisher, the editors and the reviewers. Any product that may be evaluated in this article, or claim that may be made by its manufacturer, is not guaranteed or endorsed by the publisher.
