# Supplementary material for: Testing the Functional and Phylogenetic Assembly of Plant Communities in Gobi Deserts of Northern Qinghai–Tibet Plateau
Source: Front Plant Sci. 2022 Jul 18;13:952074. doi: 10.3389/fpls.2022.952074 (PMC9340061; doi:10.3389/fpls.2022.952074)
Supplement: Supplementary file 1 [file Data_Sheet_1.doc]

**Testing the functional and phylogenetic assembly of plant communities in Gobi deserts of northern Qinghai-Tibet Plateau**

Jianming Wang^1,3^**^*^**, Yin Wang^1^, Mengjun Qu^1^, Yiming Feng^2^, Bo Wu^2^, Qi Lu^2^, Nianpeng He^3^, Jingwen Li**^1*^**

1. School of Ecology Nature Conservation, Beijing Forestry University, Beijing, China.

2. Institute of Desertification Studies, Chinese Academy of Forestry, Beijing, China.

3. Key Laboratory of Ecosystem Network Observation and Modeling, Institute of Geographic Sciences and Natural Resources Research, Chinese Academy of Science, Beijing 100101，China

**Table S1 Plant lists occurring in 61 Gobi survey sites**

|  | **Species** | **Family** | **Genera** |
| --- | --- | --- | --- |
| 1 | *Sarcozygium xanthoxylum* | Zygophyllaceae | Sarcozygium |
| 2 | *Salsola tragus* | Amaranthaceae | Salsola |
| 3 | *Stipa tianschanica var. gobica* | Poaceae | Stipa |
| 4 | *Ajania fruticulosa* | Asteraceae | Ajania |
| 5 | *Sympegma regelii* | Amaranthaceae | Sympegma |
| 6 | *Gymnocarpos przewalskii* | Caryophyllaceae | Gymnocarpos |
| 7 | *Ephedra przewalskii* | Ephedraceae | Ephedra |
| 8 | *Krascheninnikovia ceratoides* | Amaranthaceae | Krascheninnikovia |
| 9 | *Bassia dasyphylla* | Amaranthaceae | Bassia |
| 10 | *Halogeton glomeratus* | Amaranthaceae | Halogeton |
| 11 | *Nitraria tangutorum* | Nitrariaceae | Nitraria |
| 12 | *Reaumuria soongarica* | Tamaricaceae | Reaumuria |
| 13 | *Asterothamnus centraliasiaticus* | Asteraceae | Asterothamnus |
| 14 | *Nitraria sphaerocarpa* | Nitrariaceae | Nitraria |
| 15 | *Haloxylon ammodendron* | Amaranthaceae | Haloxylon |
| 16 | *Calligonum mongolicum* | Polygonaceae | Calligonum |
| 17 | *Achnatherum splendens* | Poaceae | Achnatherum |
| 18 | *Kalidium foliatum* | Amaranthaceae | Kalidium |
| 19 | *Phragmites australis* | Poaceae | Phragmites |
| 20 | *Caryopteris mongholica* | Lamiaceae | Caryopteris |
| 21 | *Salsola arbuscula* | Amaranthaceae | Salsola |
| 22 | *Artemisia desertorum* | Asteraceae | Artemisia |
| 23 | *Tamarix chinensis* | Tamaricaceae | Tamarix |
| 24 | *Tamarix ramosissima* | Tamaricaceae | Tamarix |
| 25 | *Corethrodendron multijugum* | Fabaceae | Corethrodendron |
| 26 | *Salsola laricifolia* | Amaranthaceae | Salsola |
| 27 | *Artemisia annua* | Asteraceae | Artemisia |
| 28 | *Askellia flexuosa* | Asteraceae | Askellia |
| 29 | *Sphaerophysa salsula* | Fabaceae | Sphaerophysa |
| 30 | *Scorzonera divaricata* | Asteraceae | Scorzonera |
| 31 | *Apocynum pictum* | Apocynaceae | Apocynum |
| 32 | *Lycium ruthenicum* | Solanaceae | Lycium |
| 33 | *Halostachys caspica* | Amaranthaceae | Halostachys |
| 34 | *Reaumuria kaschgarica* | Tamaricaceae | Reaumuria |
| 35 | *Myricaria bracteata* | Tamaricaceae | Myricaria |
| 36 | *Agropyron cristatum* | Poaceae | Agropyron |
| 37 | *Nitraria roborowskii* | Nitrariaceae | Nitraria |
| 38 | *Limonium aureum* | Plumbaginaceae | Limonium |
| 39 | *Calligonum caput-medusae* | Polygonaceae | Calligonum |
| 40 | *Tamarix hispida* | Tamaricaceae | Tamarix |
| 41 | *Astragalus mongholicus* | Fabaceae | Astragalus |
| 42 | *Echinops gmelinii* | Asteraceae | Echinops |
| 43 | *Salix cheilophila* | Salicaceae | Salix |
| 44 | *Halocnemum strobilaceum* | Amaranthaceae | Halocnemum |

**Table S2** Best predictor for the functional and phylogenetic assembly of plant communities in Gobi deserts of northern Qinghai-Tibet Plateau.

Soil total nitrogen content, TSN; Soil organic carbon content, TOC; Soil moisture, SM; precipitation seasonality, PS; mean annual precipitation, MAP; temperature seasonality, TS; mean annual temperature, MAT. Phylogeny, phylogenetic assembly; All traits, combine of all functional traits; Leaf nitrogen concentration, LNC; Leaf phosphorus concentration, LPC; Specific leaf area, SLA; Leaf area, LA; Root nitrogen concentration, RNC; Root phosphorus concentration, RPC; Specific root length, SRL; Root length, RL.

|  | **Local factors** | | | | | **Climatic factors** | | | | **Model *R*^2^** | **Model *P*** |
| --- | --- | --- | --- | --- | --- | --- | --- | --- | --- | --- | --- |
|  | **SM** | **TSN** | **TOC** | **pH** | **GC** | **MAT** | **TS** | **MAP** | **PS** |
| Phylogeny |  |  |  |  | 0.55 |  |  |  | -0.43 | 0.140 | <0.001 |
| All traits | 0.32 |  |  |  | -0.26 |  | -0.17 |  | 0.17 | 0.403 | <0.001 |
| LNC |  |  |  | -0.28 |  | -0.15 |  |  |  | 0.077 | <0.001 |
| LPC |  |  |  | -0.37 |  | -0.41 |  |  | 0.20 | 0.326 | <0.001 |
| SLA |  | 0.19 |  | 0.18 | -0.25 | 0.15 |  |  |  | 0.188 | <0.001 |
| LA |  |  |  | 0.23 | -0.18 |  |  | 0.10 |  | 0.363 | <0.001 |
| RNC |  |  | 0.14 |  |  |  |  |  | -0.21 | 0.088 | <0.001 |
| RPC |  |  |  |  |  | 0.62 |  |  |  | 0.299 | <0.001 |
| SRL | 0.29 |  |  |  | -0.26 |  |  | 0.16 | 0.13 | 0.378 | <0.001 |
| RL |  |  |  | 0.26 | -0.19 |  |  |  | -0.24 | 0.191 | <0.001 |


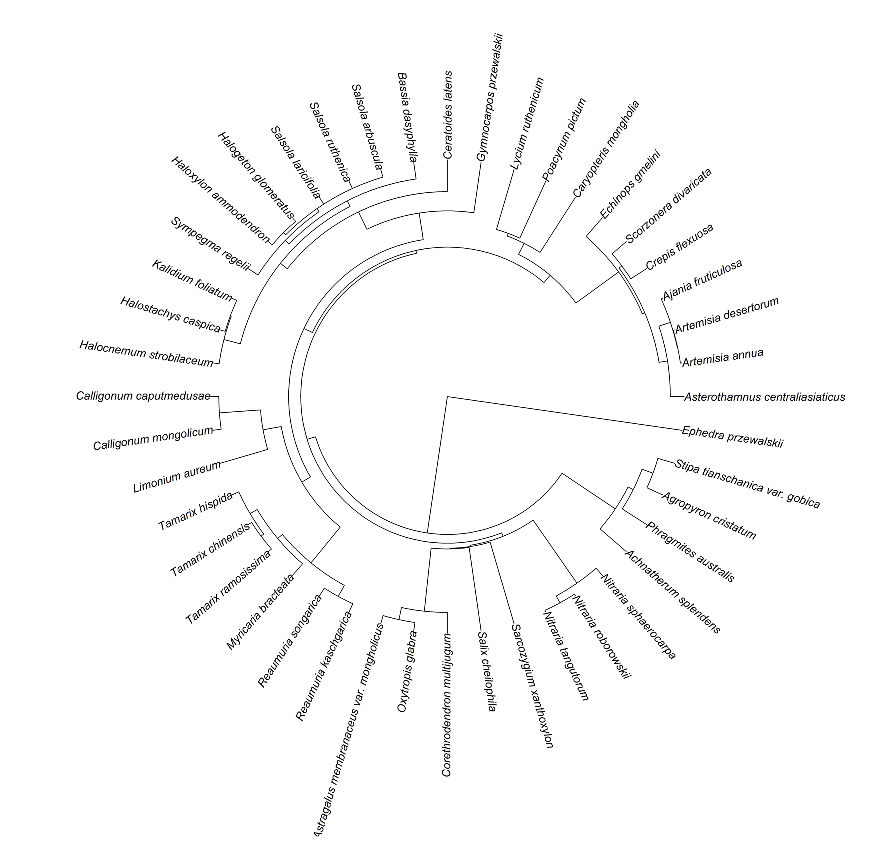


**Figure S1** Phylogenetic tree of 44 species in this study


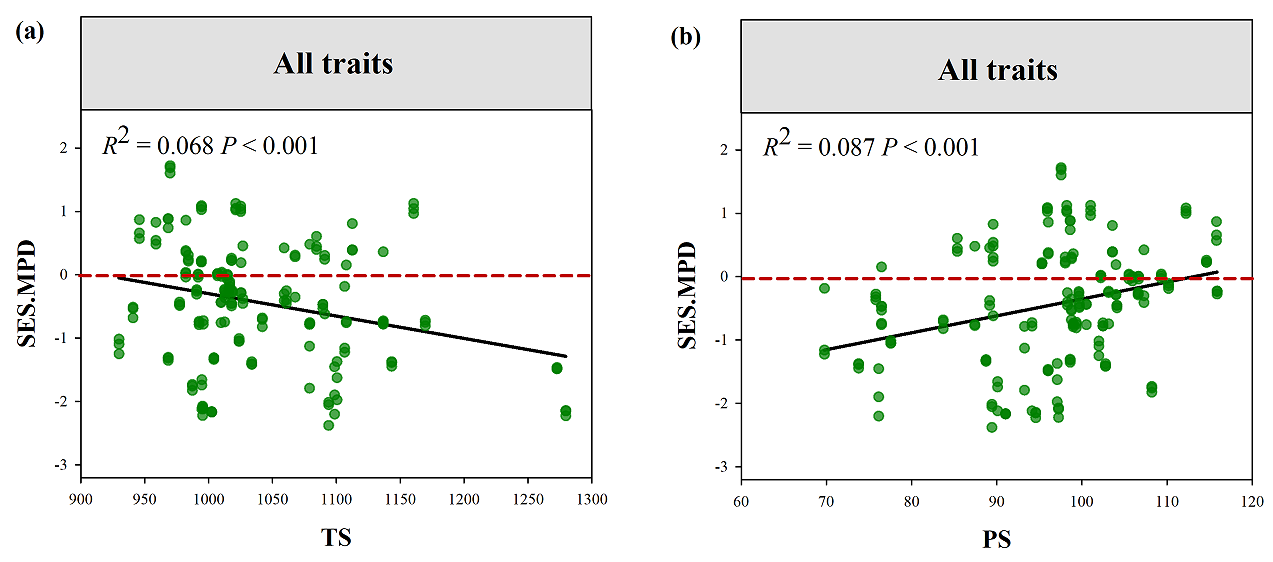


**Figure S2** Variation in standard effect size of MPD for all traits along the gradients of TS and PS


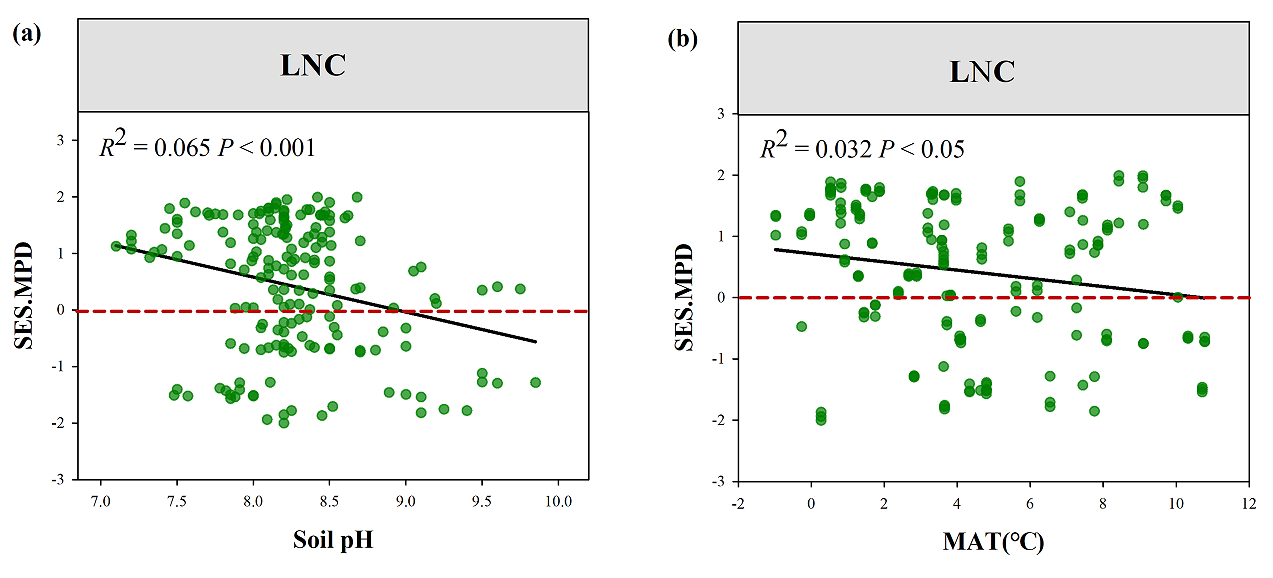


**Figure S3** Variation in standard effect size of MPD for LNC along the gradients of soil pH and MAT


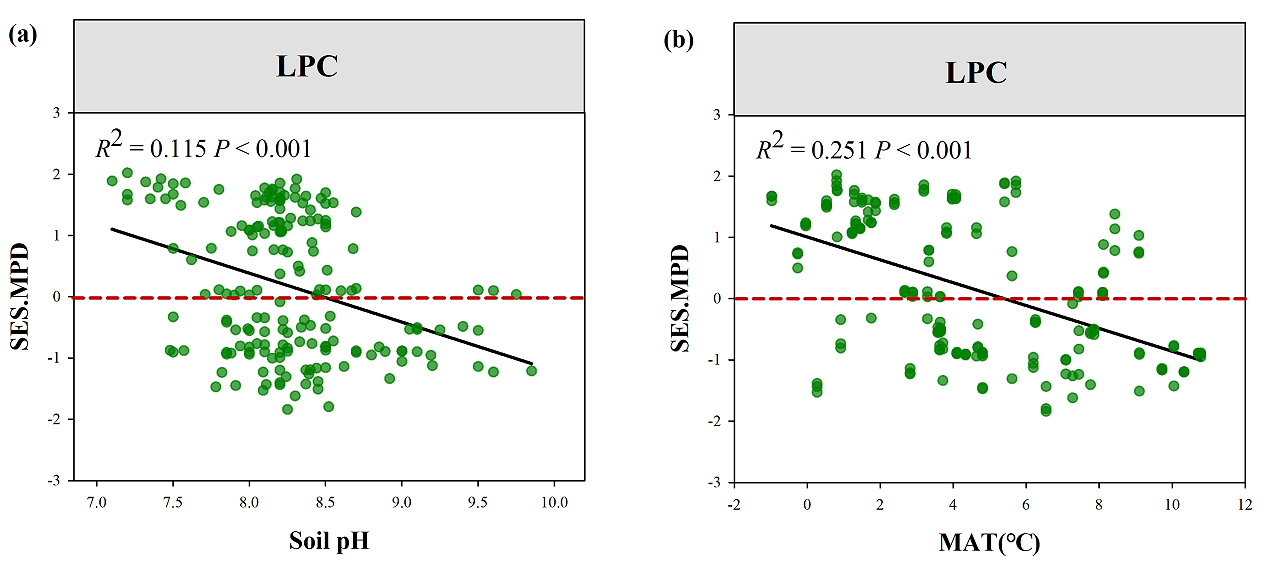


**Figure S4** Variation in standard effect size of MPD for LPC along the gradients of soil pH and MAT


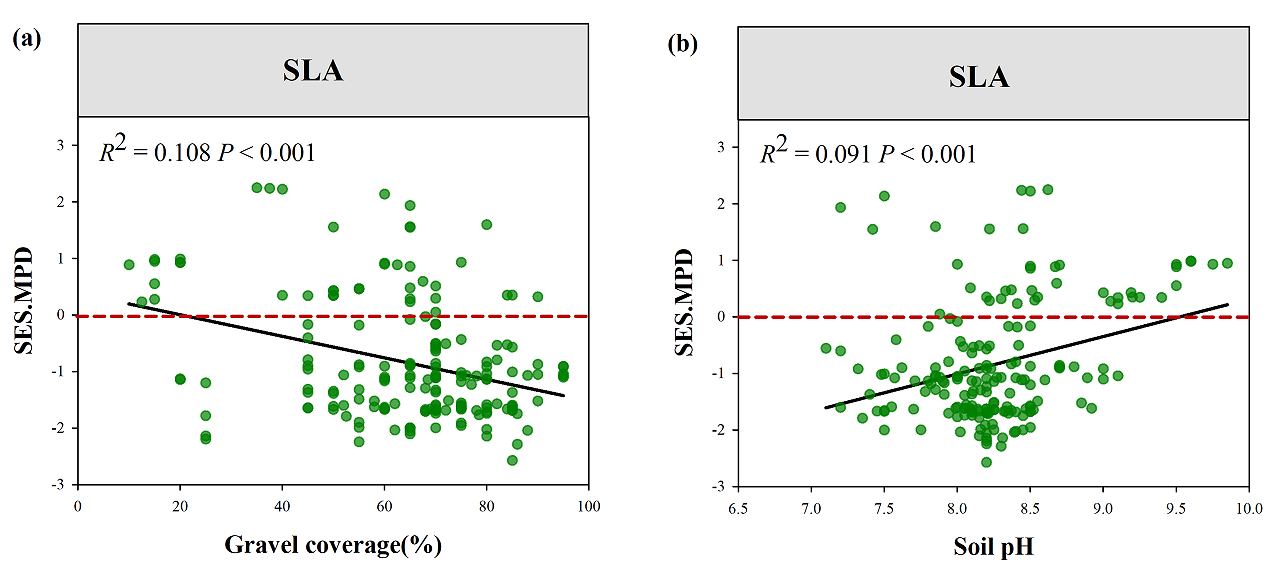


**Figure S5** Variation in standard effect size of MPD for SLA along the gradients of Gravel and soil pH


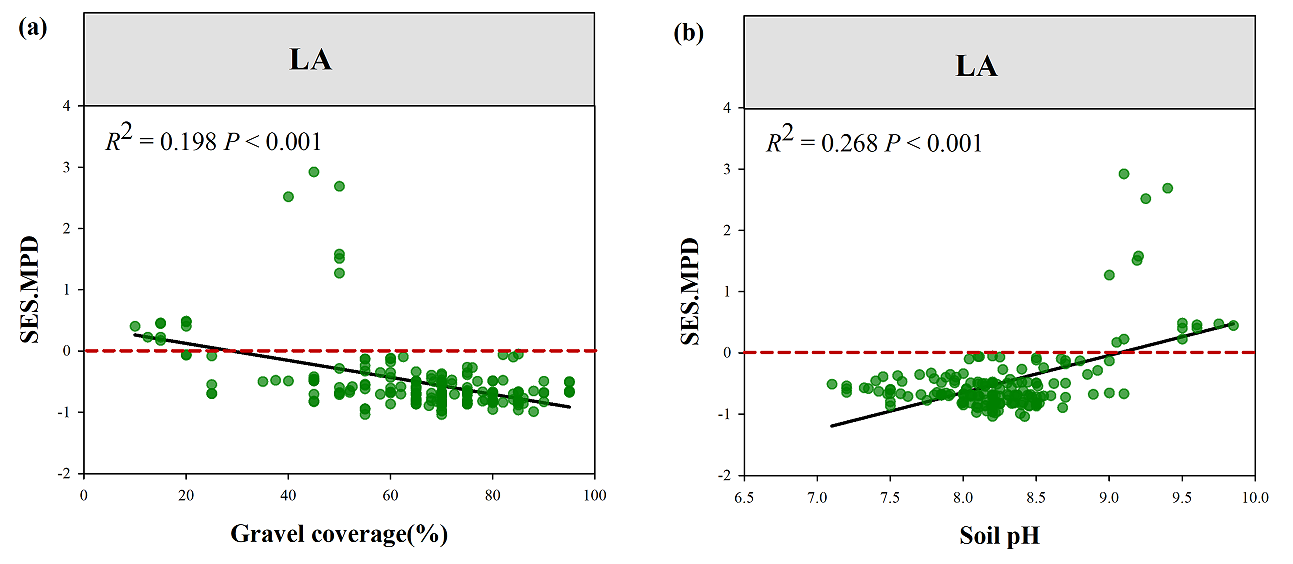


**Figure S6** Variation in standard effect size of MPD for LA along the gradients of Gravel and soil pH


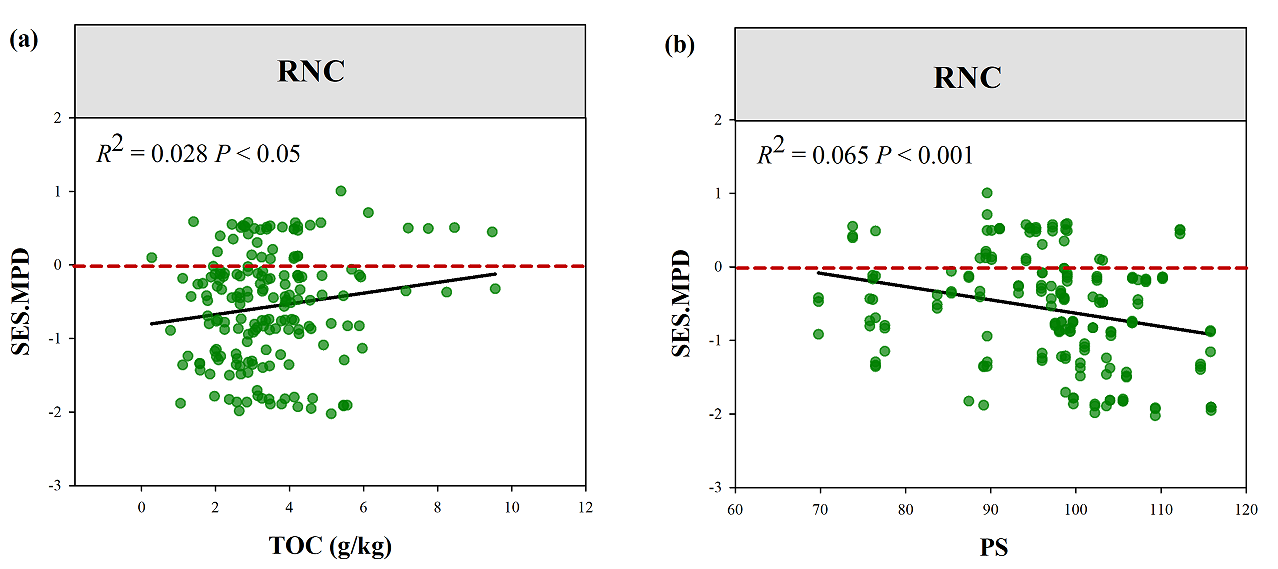


**Figure S7** Variation in standard effect size of MPD for RNC along the gradients of TOC and PS


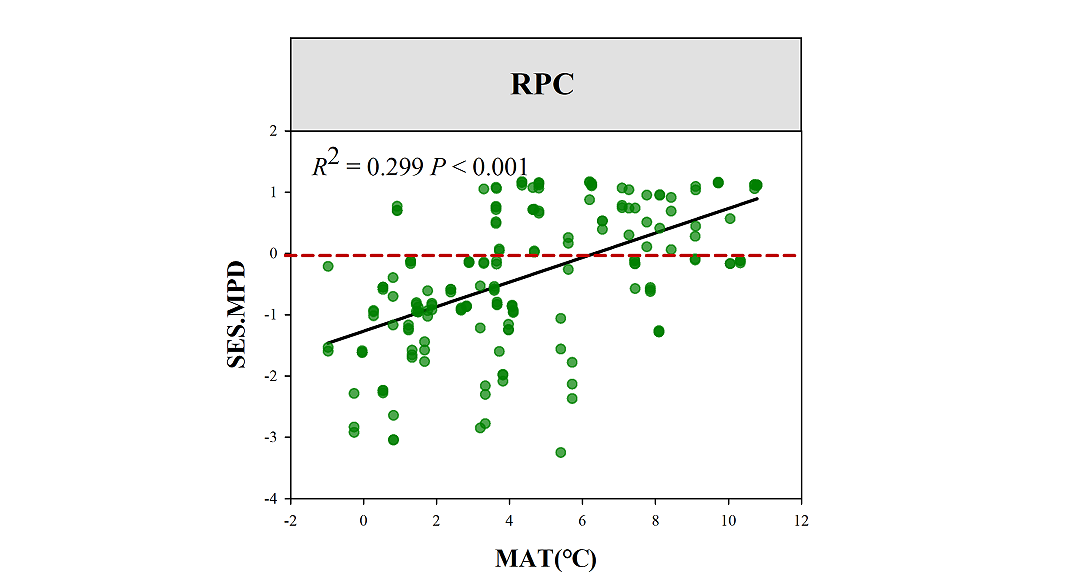


**Figure S8** Variation in standard effect size of MPD for RPC along the gradient of MAT


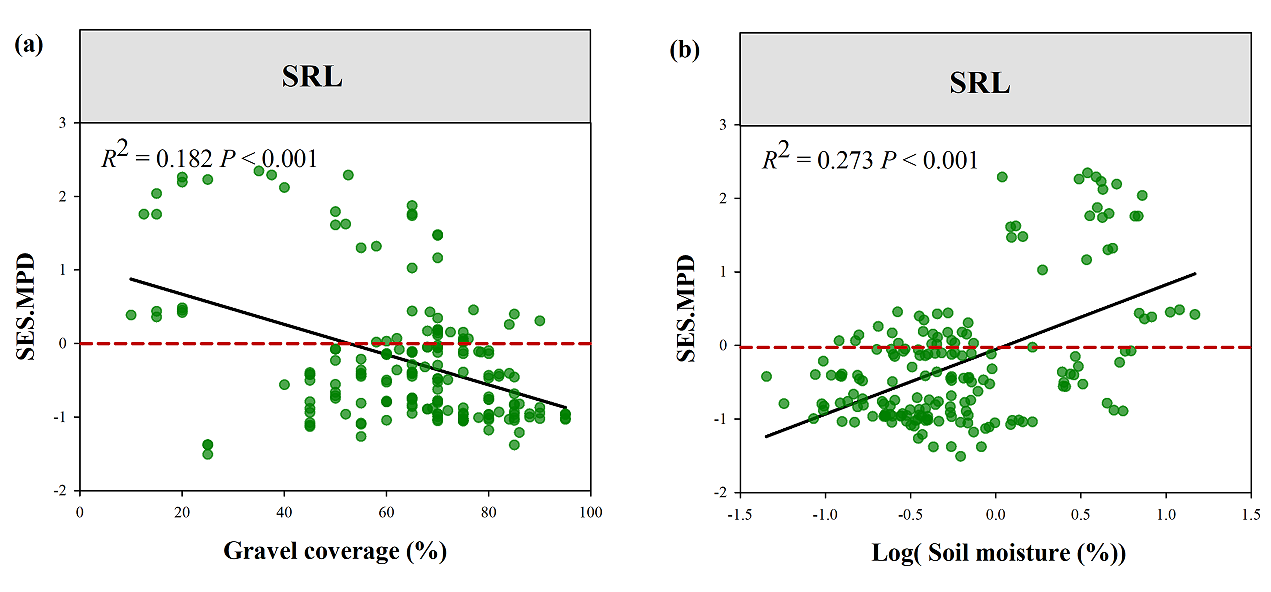


**Figure S9** Variation in standard effect size of MPD for SRL along the gradients of Gravel and SM


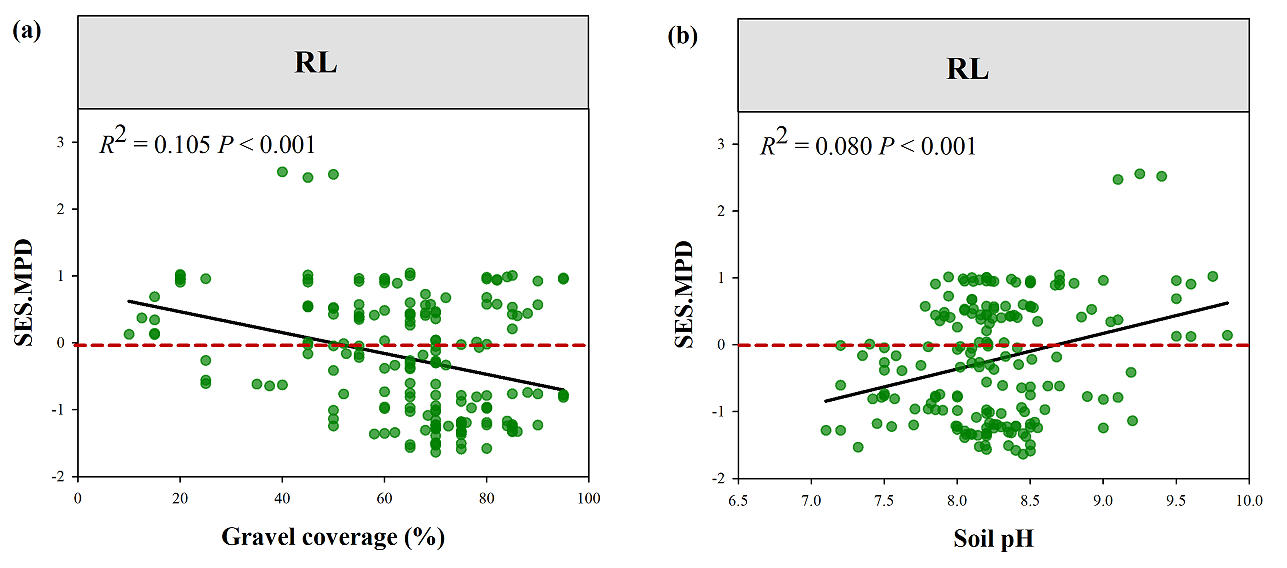


**Figure S10** Variation in standard effect size of MPD for RL along the gradients of Gravel and soil pH


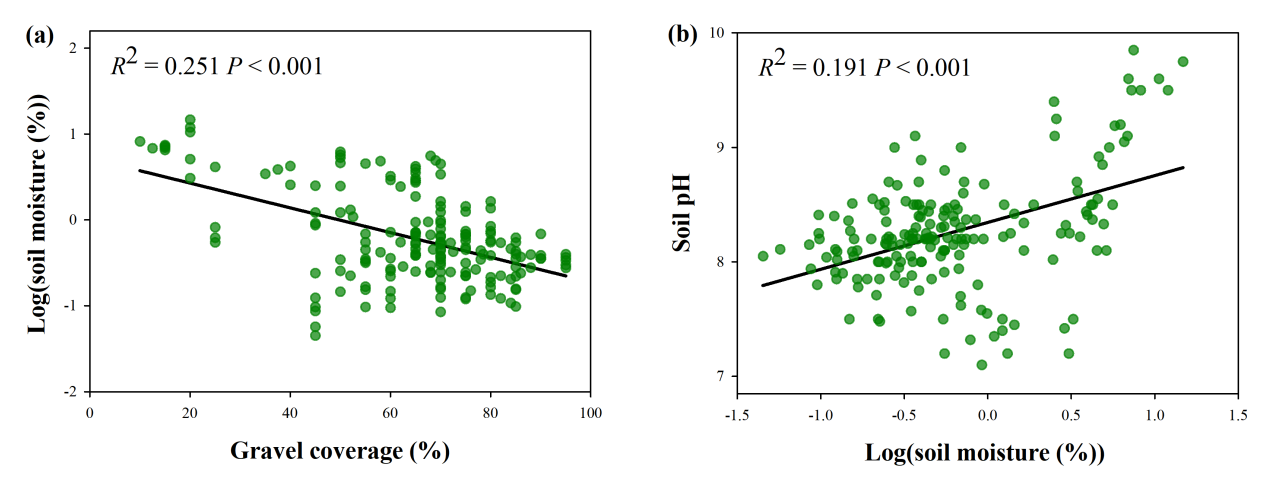


**Figure S11** Relationships between soil moisture and gravel coverage, soil pH in Gobi deserts.
